# NELD-EC: Neighborhood-Effective-Line-Density-Based Euclidean Clustering for Point Cloud Segmentation

**DOI:** 10.3390/s25041174

**Published:** 2025-02-14

**Authors:** Zhigang Su, Shixing Du, Jingtang Hao, Bing Han, Peng Ge, Yue Wang

**Affiliations:** 1 Sino-European Institute of Aviation Engineering, Civil Aviation University of China, Tianjin 300300, China; du.shix@foxmail.com (S.D.); jthao@cauc.edu.cn (J.H.); b-han@cauc.edu.cn (B.H.); 2 The 38th Research Institute of China Electronics Technology Group Corporation, Hefei 230093, China; gepeng09@gmail.com; 3 Information Countermeasure Technology Laboratory, Beijing Research Institute of Telemetry, Beijing 100076, China; wangyue@brit.com.cn

**Keywords:** lidar, point cloud clustering, effective neighborhood, adaptive threshold, uneven density

## Abstract

For the problem that it is difficult to effectively cluster lidar point clouds with irregular shapes and uneven densities, a Neighborhood Effective Line Density (NELD)-based Euclidean Clustering (NELD-EC) algorithm is proposed in this paper. The NELD-EC algorithm first eliminates the interfering points within the neighborhood of the data point by utilizing the distance relationship and calculates the NELD of the data point using the effective neighborhood set without interfering points of the data point. The NELD of a data point is taken as the local density of that data point. Then, the NELD-EC algorithm conducts clustering processing using the NELD of all data points and uses the reciprocal of the harmonic average of the local densities of all data points within each cluster after clustering as the distance threshold for the data points within the cluster. Finally, the NELD-EC algorithm completes the clustering of the point cloud based on the adjusted adaptive distance threshold. The clustering experimental results on simulated point clouds, fixed point clouds, and sequential point clouds indicate that, compared with several other typical Euclidean clustering algorithms, the NELD-EC algorithm requires simpler parameters to be set, is less sensitive to the initial distance threshold, can effectively reduce the occurrence probabilities of over-segmentation and under-segmentation, and has strong stability in clustering performance. The NELD-EC algorithm is more suitable for processing sequential point clouds in actual dynamic and complex scenarios.

## 1. Introduction

Lidar, an active detection device with high precision and long-range detection capabilities, has been widely applied in fields such as remote sensing, autonomous driving, unmanned aerial vehicle mapping, and robot navigation [1,2]. The extremely narrow needle-shaped beam employed by laser radar causes the target information it collects to present in the form of point clouds [3]. For applications based on lidar, such as target recognition and tracking, it is necessary to separate the point clouds belonging to different targets, that is, to conduct clustering processing on the collected point clouds. Due to the variations in target reflection characteristics and morphologies, point clouds exhibit irregular shapes and non-uniform densities [4,5], especially the point clouds collected that reflect large-scale environments [2,6], where there are significant density differences between the point clouds of distant and near targets. How to achieve effective clustering of irregular and non-uniform point clouds is a challenging issue in the field of point cloud processing.

At present, the methods for point cloud clustering mainly comprise model-based methods [7,8], density-based methods [5,9,10], distance-based methods [11,12,13,14], and deep learning-based methods [15,16,17,18,19]. Among these clustering methods, the distance-based methods possess the features of simple logic, fast running speed, and wide application scope, and have garnered extensive attention. The Euclidean Clustering (EC) algorithm is a classic clustering method based on Euclidean distance [11,20,21]. This method determines the neighborhood of data points according to the distance threshold and regards data points within the same neighborhood as belonging to the same category, thereby achieving the clustering of point clouds. The magnitude of the distance threshold adopted by the EC algorithm influences the clustering effect of the algorithm. An overly large or small distance threshold will cause the EC algorithm to undergo under- or over-segmentation during the clustering process. When clustering irregular and non-uniform point clouds, it is quite challenging for the EC algorithm to employ a fixed distance threshold to simultaneously avoid the occurrence of under- and over-segmentation.

To achieve effective clustering of irregular and uneven point clouds, some approaches for dynamically adjusting the distance threshold of the EC algorithm have been put forward [12,13,14,22,23], mainly including classification methods and adaptive methods. The classification method divides point cloud data into several regions based on the characteristic of points being denser in the near range and sparser in the far range according to the distance. Different distance thresholds are assigned to each region [12,22], or the boundary regions of multi-cluster point clouds are classified [23]. To a certain extent, the classification method alleviates the clustering problem of uneven point clouds. However, this method is highly directive. For point clouds that do not conform to the characteristic of being denser in the near range and sparser in the far range, this type of method cannot effectively partition the data, thus leading to poor clustering performance. The adaptive method determines the distance threshold based on the distribution of each point in the neighborhood of the data point [13,14]. The adaptive method uses the local or relative features of the data point to dynamically adjust the distance threshold, such as the LDT-EC algorithm that employs the local density-based threshold (LDT) [13], and the RDT-EC algorithm that uses the relative distance-based threshold (RDT) [14]. The EC algorithm with an adaptive threshold can adapt to the clustering problem of point clouds with different densities. These adaptive methods for determining the distance threshold all require the use of neighborhood information. The neighborhood of the data point is determined by the initial distance threshold. Therefore, the quality of the selection of the initial distance threshold will also affect the subsequent determination of the adaptive distance threshold and indirectly influence the clustering performance of the improved EC algorithm.

To achieve effective clustering of sparse and uneven point clouds, an effective approach for defining the local density of data points is proposed in this paper. This method eliminates interfering points by exploiting the distance variations among data points within the neighborhood, thereby calculating the local density of data points using only valid points. This density is defined as the Neighborhood Effective Line Density (NELD). As NELD is not influenced by interfering points, it offers a more rational characterization of the local density of data points. Based on the NELD of all data points obtained, a Euclidean clustering method with an adaptive threshold is presented, which is referred to as the NELD-based Euclidean Clustering (NELD-EC) algorithm. The NELD-EC algorithm builds upon the classic EC algorithm [11] and incorporates the idea of the LDT-EC algorithm in adaptively determining the threshold using local density. The NELD-EC algorithm partitions the point cloud into several sets based on the disparities in local density, and the reciprocal of the harmonic average of the local density of each data point within each set serves as the distance threshold for each point within that set. The NELD-EC algorithm employs these adjusted distance thresholds to carry out clustering on irregular and uneven point clouds. The main innovations of the NELD-EC algorithm lie in:

1. The NELD-EC algorithm regards the cluster of points closest to the data point within the neighborhood as the effective neighborhood points, while the others are treated as interference points. The NELD-EC algorithm utilizes the distance variance of data points within the neighborhood to eliminate the interference points and only employs the effective neighborhood points to compute the local density of the data points. Hence, this local density is insensitive to the selection of the initial distance threshold.
2. The NELD-EC algorithm partitions the point cloud into several sets in accordance with the local density variance. Points with similar densities are categorized into the same set, and the distance threshold for the data points in each set will be determined based on the local density of the points within that set. The distance threshold adjustment strategy of the NELD-EC algorithm can well address the issue of adaptive setting of distance thresholds for point clouds with different densities and enhance the clustering performance of the NELD-EC algorithm on non-uniform point clouds.

The remaining sections of this paper are structured as follows: Section 2 elaborates in detail the two key points of the density-inhomogeneous point cloud clustering task in this paper and describes and analyzes the existing related methods. The proposed NELD-EC algorithm is presented in Section 3. In Section 4, comparative experiments are carried out using simulated data sets, point cloud data collected at fixed points, and sequential point cloud data. It is proved that the proposed algorithm is significantly superior to other classic EC algorithms in terms of initial distance threshold sensitivity, suppression of under-segmentation and over-segmentation capabilities, and clustering effect. The NELD-EC algorithm demonstrates superior performance in clustering irregular and inhomogeneous point clouds. Finally, the conclusions of this paper are presented in Section 5.

## 2. Description of the Problem

Assume that the point cloud is a set composed of *I* data points in space, denoted as $P=\{p_{1},p_{2},\ldots ,p_{I}\}$. Commonly, the point cloud in space is not uniformly distributed but presents a certain aggregation form, that is, the data points are distributed in clusters. Therefore, the point cloud set *P* can also be expressed as $P=\{C_{1},C_{2},\ldots ,C_{M}\}$, where *M* is the total number of clusters in the point cloud set *P*, $C_{m}$ represents the data point set constituting the *m*-th cluster, denoted as $C_{m}=\left\{p_{m,1},p_{m,2},\ldots ,p_{m,I_{m}}\right\}$, and $I_{m}=\left|C_{m}\right|$ is the number of data points in the set $C_{m}$. The function $\left|\;\cdot \;\right|$ indicates the number of data points in the set. The clustering of the point cloud is to correctly assign each data point in the point cloud set *P* to the corresponding cluster set.

When clustering irregular and non-uniform point clouds, EC-type algorithms are required to adaptively adjust the distance threshold according to the distribution of data points in the neighborhood of the data point. For instance, the LDT-EC algorithm determines the corresponding distance threshold based on the local density of the data point. Consequently, the performance of EC-type algorithms is influenced by the definition of the local density of the data point and the method of calculating the adaptive distance threshold.

### 2.1. Definition of the Local Density of Data Point

There exist multiple definitions regarding the local density of a data point, among which the more mainstream ones fall into two categories: the definitions based on neighborhood and those based on nearest neighbors. The definition based on neighborhood takes the data point $p_{i}$ as the center and a distance threshold *d* as the radius to determine the neighborhood, and the points within this area constitute the neighborhood set of $p_{i}$

(1) $$S_{d}\left(p_{i}\right)=\left\{p_{j}|d_{ij}\leq d\right\}$$

where $d_{ij}$ denotes the Euclidean distance between two points, $p_{i}$ and $p_{j}$, in space. For the convenience of subsequent descriptions, $d_{ij}$ is referred to as the $p_{j}$-distance of point $p_{i}$. According to the neighborhood set $S_{d}\left(p_{i}\right)$ of point $p_{i}$, the local density of point $p_{i}$ can be defined as

(2) $$\rho_{i}=\frac{\left|S_{d}\left(p_{i}\right)\right|}{4\pi d^{3}/3}$$

from Equation (2), the local density of data points based on the neighborhood definition refers to the number of data points per unit volume within the data point’s neighborhood.

Another category of methods for determining the data point set around $p_{i}$ is to employ the proximity relationship among data points. Commonly used proximity relationships include K-nearest neighbors (KNN), reverse K-nearest neighbors (RNN), etc. The set of K-nearest neighbors of data point $p_{i}$ is defined as

(3) $$S_{\mathrm{KNN}}\left(p_{i}\right)=\left\{p_{j}|d_{ij}\leq d_{i}^{K}\right\}$$

where $d_{i}^{K}$ indicates the *K*-th smallest $p_{j}$-distance from data point $p_{i}$. By utilizing the K-nearest neighbor set $S_{\mathrm{KNN}}\left(p_{i}\right)$, the local density of data point $p_{i}$ can be defined as

(4) $$\rho_{i}=\frac{\left|S_{\mathrm{KNN}}\left(p_{i}\right)\right|}{\sum_{p_{j}\in S_{\mathrm{KNN}}\left(p_{i}\right)}d_{ij}}$$

Evidently, the local density defined by Equation (4) can better depict the variations in local density resulting from different distribution patterns of the same number of points near point $p_{i}$. On the foundation of Equation (4), successive improved versions of various local density definitions have been presented [24,25]. These enhanced versions either modify the calculation method of the denominator in Equation (4) or adopt a way of rapidly constructing the neighborhood set, thereby attaining a more rational description of the local density of data point $p_{i}$.

**Figure 1:**
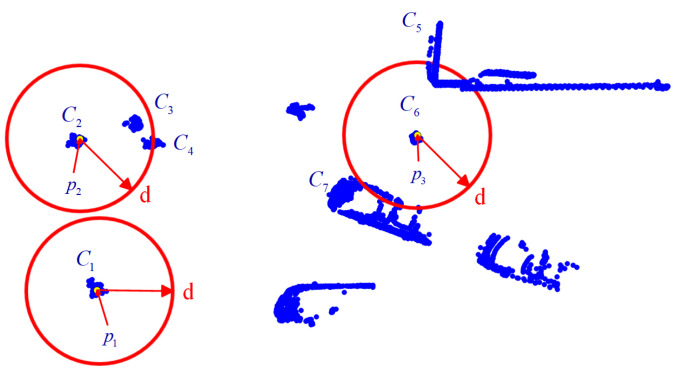
Top view of a portion of the 3D point cloud at an intersection in the Sydney urban area.

By comparing Equation (1) with Equation (3), it can be observed that the neighbor-hood set $S_{d}\left(p_{i}\right)$ determined by Equation (1) employs an identical radius for any data point within the point cloud, whereas the K-nearest neighbor set $S_{\mathrm{KNN}}\left(p_{i}\right)$ defined by Equation (3) involves a radius that varies with the selected data point. Nevertheless, both the neighborhood set and the nearest neighbor set are centered on the data point $p_{i}$ and determine the scope of data points encompassed by the set within a certain radius. This approach of determining nearby points inevitably causes some interfering points to be incorporated into the set. Taking the three-dimensional point cloud data of a certain intersection in the urban area of Sydney as an example, its top view is depicted in Figure 1. As shown in Figure 1, the point cloud data on the horizontal plane consists of multiple clusters. For a given distance threshold *d*, the ideal neighborhood set should be similar to the situation around data point $p_{1}$ in the figure. However, in actual data, circumstances similar to those of $p_{2}$ and $p_{3}$ are bound to exist. As illustrated in Figure 1, the clusters $C_{3}$ and $C_{4}$ within the neighborhood of point $p_{2}$, and the clusters $C_{5}$ and $C_{7}$ within the neighborhood of point $p_{3}$, interfere with the local density calculation of data points $p_{2}$ and $p_{3}$, thereby influencing the subsequent clustering processing of the point cloud. Hence, it is necessary to explore more rational local density calculation methods to realize the elimination of the influence of interfering points on the local density of the data point.

### 2.2. Determination of the Adaptive Distance Threshold

In EC-type algorithms, the adaptive distance threshold can be determined either based on the local density or relative distance.

The LDT-EC algorithm adaptively determines the distance threshold of data points based on local density. The LDT-EC algorithm computes the local density $\rho_{i}$ of all data points in accordance with the initial distance threshold *d* by Equation (2) and acquires the local density mean $\bar{\rho }$. The LDT-EC algorithm ascertains the adaptive distance threshold based on the magnitude relationship between the local density $\rho_{i}$ of the data point and the local density mean $\bar{\rho }$:

(5) $$d_{i}^{+}=\left\{\begin{array}{cc} \sum_{p_{j}\in S_{d}\left(p_{i}\right)}\frac{d_{ij}}{|S_{d}\left(p_{i}\right)|} & \rho_{i}\geq \bar{\rho }\\ \max\{d_{ij}|p_{j}\in S_{d}\left(p_{i}\right)\} & \rho_{i}<\bar{\rho } \end{array}\right.$$

It is evident from Equation (5) that for data points with a relatively high local density, their distance threshold $d_{i}^{+}$ is determined by the mean of the $p_{j}$-distances within the neighborhood of that point. However, for data points with a relatively low local density, their distance threshold $d_{i}^{+}$ is defined by the maximum $p_{j}$-distance within the neighborhood of the point. Clearly, the distance threshold $d_{i}^{+}$ determined by Equation (5) is associated with the local density of data point $p_{i}$ and the distribution of data points within its neighborhood. As the distribution of data points within the neighborhood of data point $p_{i}$ changes, the distance threshold $d_{i}^{+}$ also adaptively varies. As the local density of data points is influenced by the initial distance threshold, consequently, the selection of the initial distance threshold indirectly affects the adaptive distance threshold $d_{i}^{+}$.

The RDT-EC algorithm employs the relative distance between data points and the lidar to define the adaptive distance threshold [14].

(6) $$d_{i}^{+}=\alpha \frac{d_{i}}{d_{0}}d$$

where $d_{0}$ indicates the Euclidean distance between the lidar and the nearest data point in the point cloud, $d_{i}$ is the distance between the data point $p_{i}$ and the lidar, and α is the adjustment parameter.

From the above analysis, it is evident that regardless of whether it is the method based on local density or the one based on relative distance, the setting of the adaptive distance threshold relies on the selection of the initial distance threshold. The quality of the initial distance threshold selection influences the determination of the adaptive distance threshold and further affects the clustering effect of the point cloud.

## 3. NELD-EC Algorithm

Given that the initial distance threshold has an impact on the local density of data points and the adaptive distance threshold in the EC-type algorithms, in this section, the method is studied to mitigate the influence of the initial distance threshold, and subsequently, the NELD-EC algorithm is proposed for irregular and uneven point cloud clustering.

### 3.1. Elimination of the Interfering Points Within the Neighborhood

As previously stated, the existence of interfering points within the neighborhood of a data point affects the value of the local density of the data point. With the increase in the distance threshold, the number of interfering points within the neighborhood will also increase, thereby weakening the descriptive ability of the local density for the data point. Hence, to reduce the influence of the initial distance threshold on the local density, it is necessary to eliminate the interfering points within the neighborhood.

It can be observed from the example in Figure 1 that the interfering points within the neighborhood of the data point are usually farther away from the data point compared to the valid points. Therefore, the interfering points can be distinguished from the valid points from the perspective of distance.

For the data point $p_{i}$, given the initial distance threshold *d*, the neighborhood set $S_{d}\left(p_{i}\right)$ can be acquired. The distance $d_{ij}$ between the data point $p_{i}$ and each data point $p_{j}$ within the neighborhood set $S_{d}\left(p_{i}\right)$ is calculated. Without loss of generality, the distances $d_{ij}$ of each data point are quantified with the unit of the smallest non-zero distance

(7) $$\delta =\min_{d_{ij}>0}d_{ij}$$

constituting the neighborhood distance set

(8) $$D\left(p_{i}\right)=\left\{d_{ij}|p_{j}\in S_{d}\left(p_{i}\right)\right\}$$

of the data point $p_{i}$.

**Figure 2:**
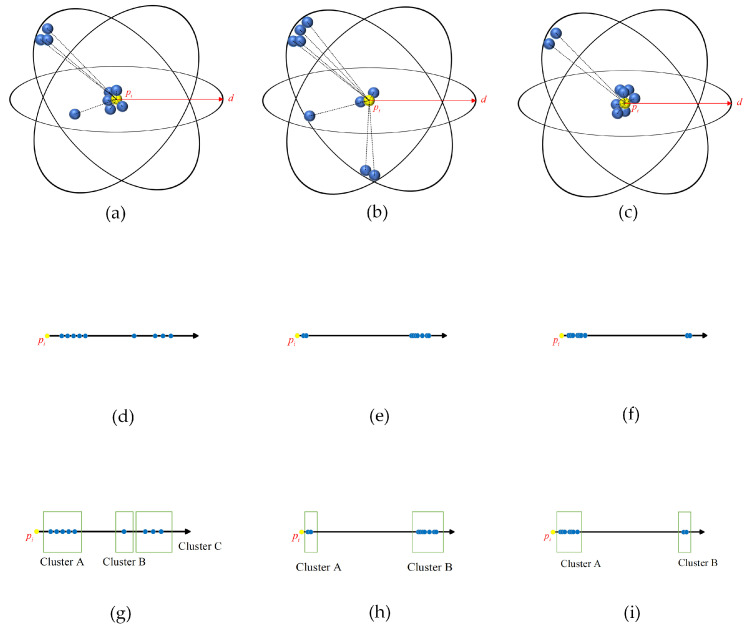
Spatial distribution of neighborhood data points containing interfering points: (**a**) Regular case. (**b**) Case with a large number of interfering points. (**c**) Case with a small number of interfering points. (**d**) Distribution of elements in the neighborhood distance set along the distance axis in the regular case. (**e**) Distribution of elements in the neighborhood distance set along the distance axis when a large number of interfering points are present. (**f**) Distribution of elements in the neighborhood distance set along the distance axis when a small number of interfering points are present. (**g**) Clustering results in the regular case. (**h**) Clustering results when a large number of interfering points are present. (**i**) Clustering results when a small number of interfering points are present.

Figure 2 presents three typical spatial distribution scenarios of data points $p_{i}$ when interfering points exist within their neighborhood. Figure 2a represents a relatively conventional spatial distribution, where valid points are relatively concentrated around $p_{i}$, while interfering points are distributed in regions farther away. Figure 2b and Figure 2c depict more extreme cases of neighborhood distributions, corresponding to scenarios where a relatively large and small number of interfering points are present, respectively. To establish a distance axis with data point $p_{i}$ as the origin, the elements of the set $D\left(p_{i}\right)$ are mapped onto this axis, as illustrated in Figure 2d–f. From the distribution of elements in the set $D\left(p_{i}\right)$ along the numerical axis, it can be observed that the $p_{j}$-distances within the neighborhood of data point $p_{i}$ exhibit variability. A suitable clustering method, such as the DBSCAN algorithm, can be employed to categorize the elements in $D\left(p_{i}\right)$ based on their relative distances. The clustering results for Figure 2d, Figure 2e, and Figure 2f are shown in Figure 2g, Figure 2h, and Figure 2i, respectively. A comparison of the clustering results under the three scenarios indicates that the clusters formed by interfering points are generally positioned farther from the origin, whereas the clusters composed of valid points are typically the closest to the origin.

For a given data point $p_{i}$, assuming that its corresponding neighborhood distance set $D\left(p_{i}\right)$ is clustered into $M_{D}$ clusters, the set $D\left(p_{i}\right)$ can then be expressed in the form of a cluster set as

(9) $$D\left(p_{i}\right)=\left\{C_{1}^{D},C_{2}^{D},\ldots ,C_{M_{D}}^{D}\right\}$$

where $C_{m}^{D}$ denotes the *m*-th cluster in the set $D\left(p_{i}\right)$. The cluster that is closest to the data point $p_{i}$ is selected as the effective neighborhood distance set

(10) $$D^{V}\left(p_{i}\right)=\min_{C_{m}^{D}}\frac{1}{\left|C_{m}^{D}\right|}\sum_{d_{ij}\in C_{m}^{D}}d_{ij}$$

the set composed of the data points corresponding to the effective neighborhood distance set $D^{V}\left(p_{i}\right)$ is referred to as the effective neighborhood set

(11) $$S_{d}^{V}\left(p_{i}\right)=\left\{p_{j}\left|d_{ij}\in D^{V}\left(p_{i}\right)\right.\right\}$$

the set $S_{d}^{V}\left(p_{i}\right)$ fulfills the purpose of eliminating interfering points within the neighborhood of the data point $p_{i}$.

The effective neighborhood set $S_{d}^{V}\left(p_{i}\right)$ obtained after clustering the neighborhood of data point $p_{i}$ not only eliminates the impact of interfering points on the local density but also reduces the sensitivity of subsequent processing to the initial distance threshold *d*. When the initial distance threshold *d* increases to a certain extent, the effective points have been completely encompassed within the neighborhood of the data point, and the interfering points within the neighborhood can be identified and removed through clustering. Thus, the initial distance threshold *d* no longer affects the calculation of the local density of the data point.

**Figure 3:**
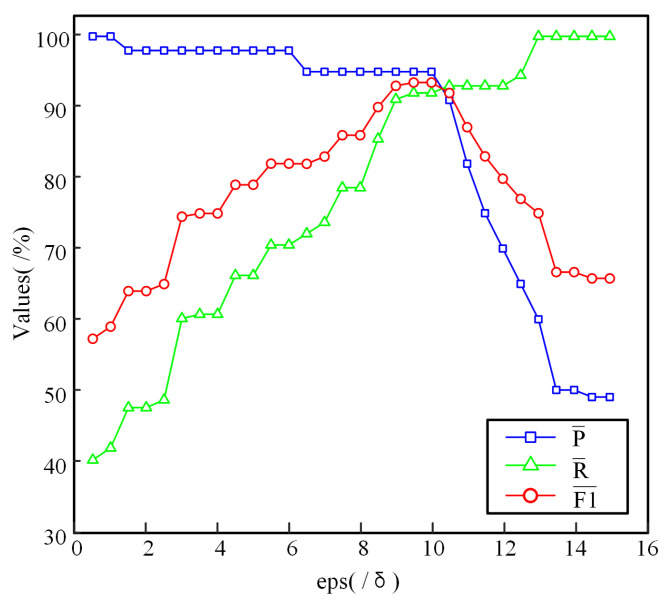
Identification performance of the valid distance set as a function of the neighborhood radius eps.

When clustering the neighborhood distance set $D\left(p_{i}\right)$, the DBSCAN algorithm requires the determination of two parameters: the minimum number of points minPts and the neighborhood radius eps. To ensure that all density-connected points are merged into the same cluster during the clustering process of $D\left(p_{i}\right)$, all points within the set $D\left(p_{i}\right)$ must be regarded as core points, and thus minPts is set to 1. The neighborhood radius eps controls the neighborhood size during DBSCAN clustering and influences the cluster boundaries, meaning that the selection of eps directly affects the ability to accurately identify the valid neighborhood distance set $D^{V}\left(p_{i}\right)$. Using 50 randomly selected data points from the three-dimensional point cloud dataset of an intersection in downtown Sydney, as shown in Figure 1, as the experimental dataset, the smallest non-zero distance δ within each data point’s neighborhood is taken as the unit of analysis to examine the variation in the average precision $\bar{P}$, recall $\bar{R}$, and F1 $\bar{F1}$ of the valid neighborhood distance set $D^{V}\left(p_{i}\right)$ under different values of eps, as illustrated in Figure 3.

As observed from Figure 3, at relatively small values of the neighborhood radius eps, the average precision remains close to 100%, indicating that the elements in the valid neighborhood distance set $D^{V}\left(p_{i}\right)$ are almost entirely composed of valid points. However, at this stage, the average recall remains at a relatively low level, suggesting that some valid points have not been clustered into $D^{V}\left(p_{i}\right)$. As eps increases, the average recall progressively rises, implying that more valid points are successfully clustered into $D^{V}\left(p_{i}\right)$. When eps reaches a relatively large value, the average recall approaches 100%, indicating that nearly all valid points have been incorporated into $D^{V}\left(p_{i}\right)$. At high recall levels, a gradual decline in average precision is observed, which suggests that an increasing number of interfering points are included in $D^{V}\left(p_{i}\right)$. In summary, excessively small or large values of eps fail to yield a reasonable valid neighborhood distance set $D^{V}\left(p_{i}\right)$, a conclusion that is further supported by the variation in the average F1 with respect to eps. As shown in Figure 3, when eps is set within 9 to 10.5 times the smallest nonzero distance δ, the average F1 remains at a relatively high level. Therefore, when applying the DBSCAN algorithm to cluster the valid neighborhood distance set $D^{V}\left(p_{i}\right)$, the optimal neighborhood radius eps is determined as 10δ.

In this paper, the method for eliminating interfering points within the neighborhood of data point $p_{i}$ is referred to as the EIP algorithm, with the detailed operational procedure presented in Algorithm 1. For a given data point $p_{i}$, the distances between each point in the neighborhood set $S_{d}\left(p_{i}\right)$ and data point $p_{i}$ are computed and quantified with the minimum non-zero distance δ to form the neighborhood distance set $D\left(p_{i}\right)$. The DBSCAN algorithm is employed to conduct clustering on the set $D\left(p_{i}\right)$. The minimum number of points, minPts, of the DBSCAN algorithm is set to 1, and the neighborhood radius, eps, is set to 10δ. After the clustering by the DBSCAN algorithm, the cluster set $\left\{C_{1}^{D},C_{2}^{D},\ldots ,C_{M_{D}}^{D}\right\}$ is formed. The cluster closest to the data point $p_{i}$ is chosen as the valid neighborhood distance set $D^{V}\left(p_{i}\right)$. The data points corresponding to all elements in the set $D^{V}\left(p_{i}\right)$ are identified to constitute the valid neighborhood set $S_{d}^{V}\left(p_{i}\right)$. Hereby, the elimination of the interfering points within the neighborhood of data point $p_{i}$ is accomplished.
**Algorithm 1** EIP algorithm**Input:** Data point $p_{i}$, and the neighborhood set $S_{d}\left(p_{i}\right)$ **Output:** The valid neighborhood set $S_{d}^{V}\left(p_{i}\right)$  1:Calculate the distances between each point within the neighborhood set $S_{d}\left(p_{i}\right)$ and the data point $p_{i}$, and construct the neighborhood distance set $D\left(p_{i}\right)$ by quantizing all the distances with the smallest non-zero distance δ. 2:Utilize the DBSCAN algorithm for clustering processing of the set $D\left(p_{i}\right)$. Set the neighborhood radius eps =10δ and the minimum number of points minpts = 1 for the DBSCAN algorithm. 3:Based on the clustering results $\left\{C_{1}^{D},C_{2}^{D},\ldots ,C_{M_{D}}^{D}\right\}$ of the DBSCAN algorithm, select the cluster that is closest to the data point $p_{i}$ as the valid neighborhood distance set $D^{V}\left(p_{i}\right)$. 4:Identify the corresponding data points for the elements of the set $D^{V}\left(p_{i}\right)$ and constitute the valid neighborhood set $S_{d}^{V}\left(p_{i}\right)$ based on them.

### 3.2. Calculation of the Local Density

The valid neighborhood set $S_{d}^{V}\left(p_{i}\right)$ can fully reflect the distribution of data points around the data point $p_{i}$, and it can be employed to compute the local density of the data point $p_{i}$. To mitigate the dependence of the local density on the initial distance threshold, the local density of the data point $p_{i}$ is calculated in the form of Equation (4), i.e.,

(12) $$\rho_{i}=\frac{\left|S_{d}^{V}\left(p_{i}\right)\right|}{\sum_{p_{j}\in S_{d}^{V}\left(p_{i}\right)}d_{ij}}$$

the local density is termed NELD in this paper. Compared with Equation (4), Equation (12) is free from the influence of interfering points and is more capable of fully depicting the distribution of data points in the vicinity of data point $p_{i}$.

For the computation of the local density of data point $p_{i}$, a special situation needs to be taken into account: there are no other data points within the neighborhood defined by the initial distance threshold *d* of data point $p_{i}$, that is, the neighborhood set $S_{d}\left(p_{i}\right)$ is an empty set. For such data points, it is advisable to set their local density directly to zero, i.e., $\rho_{i}=0$.

### 3.3. Determination of the Adaptive Distance Threshold

Calculate the local densities of all the data points of the point cloud. After normalizing with the maximum local density, a local density set is formed as

(13) $$R=\left\{\rho_{i}\left|p_{i}\in P\right.\right\}$$

perform clustering processing on the local density set R. The DBSCAN algorithm is still adopted for clustering, with the minimum number of points within the neighborhood, minpts, set as 1 and the neighborhood radius, eps, set as 0.1. Based on the local density distribution of the data points, $M_{\rho }$ clusters can be formed. It is expedient to represent the local density set R in the form of cluster classes as

(14) $$R=\{C_{1}^{\rho },C_{2}^{\rho },\ldots ,C_{M_{\rho }}^{\rho }\}$$

where the set $C_{m}^{\rho }$ denotes the *m*-th cluster formed by local densities. For the density class $C_{m}^{\rho }$, the data points within it have similar local densities. Therefore, the same adaptive distance threshold can be defined for all the data points within the set $C_{m}^{\rho }$ as

(15) $$d_{m}^{+}=\frac{1}{\left|C_{m}^{\rho }\right|}\sum_{\rho_{i}\in C_{m}^{\rho }}\rho_{i}^{-1}$$

evidently, Equation (15) employs the reciprocal of the harmonic average data of all elements in the set $C_{m}^{\rho }$ as the adaptive distance threshold for the corresponding data points.

By means of Equation (15), an appropriate adaptive distance threshold can be as-signed to each data point, making preparations for the subsequent clustering of the point cloud.

### 3.4. NELD-EC Algorithm

The NELD-EC algorithm improves the method of determining the distance threshold based on the EC algorithm. During the clustering process, the allocation strategy of data points still adopts the same strategy as the EC algorithm. The specific process of the NELD-EC algorithm is summarized as Algorithm 2.

The NELD-EC algorithm process is divided into two phases: data preparation and data point allocation. The data preparation phase is mainly constituted by steps 1 to 6 of Algorithm 2. In the data preparation phase, the neighborhood set of each data point $p_{i}$ is initially determined based on the initial distance threshold *d*, and the interfering points are eliminated from the neighborhood set to form the corresponding valid neighborhood set $S_{d}^{V}\left(p_{i}\right)$. Subsequently, the NELD of the data point is computed using the valid neighborhood set $S_{d}^{V}\left(p_{i}\right)$, and all the NELDs of the data points constitute the local density set R. The local density set R is clustered according to density, and for each density cluster, its adaptive threshold $d_{m}^{+}$ is determined and used as the adaptive distance threshold $d_{i}^{+}$ for all the data points within the cluster. Finally, the valid neighborhood set of each data point is re-determined based on its adaptive distance threshold $d_{i}^{+}$. Thus far, the data preparation for the NELD-EC algorithm has been accomplished.

Steps 7 to 16 of Algorithm 2 constitute the data point allocation phase of the NELD-EC algorithm. The data point allocation phase mainly operates on the set $P_{\mathrm{temp}}$. The set $P_{\mathrm{temp}}$ is composed of data points in the point cloud set *P* whose local density is not zero. This set is used to temporarily store the data points to be allocated in the data point allocation phase. Once the allocation of one cluster of data is completed, the data points of this cluster are deleted from the set $P_{\mathrm{temp}}$, as indicated in step 15. When all the data points in the set $P_{\mathrm{temp}}$ have been allocated, the set $P_{\mathrm{temp}}$ becomes an empty set, and the corresponding clustering process comes to an end. The clustering of each cluster is achieved through steps 9 to 14 of Algorithm 2. Select any data point $p_{i}$ from the set $P_{\mathrm{temp}}$ as the starting point of the new cluster $C_{M}$, incorporate this point and its neighborhood set $S_{d}\left(p_{i}\right)$ into $C_{M}$, and simultaneously form the temporary set $C_{\mathrm{temp}}$ from the neighborhood set $S_{d}\left(p_{i}\right)$. Subsequently, select a data point $p_{j}$ from the set $C_{\mathrm{temp}}$, and merge the data points in the neighborhood set $S_{d}\left(p_{j}\right)$ that do not belong to the set $C_{M}$ into the sets $C_{M}$ and $C_{\mathrm{temp}}$, respectively. Then, the data point $p_{j}$ is removed from the set $C_{\mathrm{temp}}$. Repeat the operations on the data points in the set $C_{\mathrm{temp}}$ until the set $C_{\mathrm{temp}}$ is an empty set. At this time, the neighborhood sets of all the data points in the set $C_{M}$ are included within the set $C_{M}$, and the clustering task for this cluster is accomplished.
**Algorithm 2** NELD-EC algorithm.**Input:** Point cloud set $P=\{p_{1},p_{2},\ldots ,p_{I}\}$, and the initial distance threshold *d* **Output:** Clusters after clustering $C_{1},C_{2},\ldots ,C_{M}$    1:Based on the initial distance threshold *d*, utilize Algorithm 1 to eliminate the interfering points within the neighborhood of each data point in the point cloud set *P*, and acquire the corresponding valid neighborhood set $S_{d}^{V}\left(p_{i}\right)$.   2:Create a duplicate of the point cloud set *P*, namely $P_{\mathrm{temp}}$, and eliminate the data points from the set $P_{\mathrm{temp}}$ whose valid neighborhood set $S_{d}^{V}\left(p_{i}\right)$ is an empty set.   3:Based on Equation (12), compute the local density of each data point in the set $P_{\mathrm{temp}}$, and normalize all the local densities with the maximum local density to construct the local density set R.   4:Using the DBSCAN algorithm, the set R is clustered, with the neighborhood radius eps = 0.1 and the minimum point count minpts = 1, resulting in the density clustering result $R=\{C_{1}^{\rho },C_{2}^{\rho },\ldots ,C_{M_{\rho }}^{\rho }\}$.   5:For each density cluster $C_{m}^{\rho }$, the adaptive threshold $d_{m}^{+}$ of this cluster is calculated by Equation (15), and this threshold is assigned to each data point $d_{i}^{+}$ within the cluster.   6:According to the adaptive distance threshold $d_{i}^{+}$ of each $p_{i}$ within the set $P_{\mathrm{temp}}$, the valid neighborhood set $S_{d}^{V}\left(p_{i}\right)$ of this data point is redefined.   7:Set the total number of clusters to zero, with M=0.   8:**while** the set $P_{\mathrm{temp}}$ is not an empty set **do**   9:     M=M+1, initialize the *M*-th cluster set $C_{M}$ and the temporary set $C_{\mathrm{temp}}$. 10:     In the set $P_{\mathrm{temp}}$, select any data point $p_{i}$ randomly. Incorporate both $p_{i}$ and its valid neighborhood set $S_{d}^{V}\left(p_{i}\right)$ into the set $C_{M}$, and simultaneously incorporate the set $S_{d}^{V}\left(p_{i}\right)$ into the set $C_{\mathrm{temp}}$. 11:     **while** the set $C_{\mathrm{temp}}$ is not an empty set **do** 12:         Choose any data point $p_{j}$ randomly from the set $C_{\mathrm{temp}}$, and add the data points in its effective neighborhood set $S_{d}^{V}\left(p_{j}\right)$ that do not belong to the set $C_{M}$ to both the set $C_{M}$ and the set $C_{\mathrm{temp}}$ respectively. 13:         Remove the data point $p_{j}$ from the temporary set $C_{\mathrm{temp}}$. 14:     **end while** 15:     Remove the data points from the temporary set $P_{\mathrm{temp}}$ that are contained in the set $C_{M}$. 16:**end while**

### 3.5. Complexity Analysis

The computational and spatial complexity of the NELD-EC algorithm is analyzed based on the details of each step.

The NELD-EC algorithm first requires performing a neighborhood search for all data points in the point cloud using the initial distance threshold. Assuming the number of data points in the spatial point cloud is *I*, the computational complexity of the neighborhood search varies depending on the implementation method. When using a brute-force search approach, the computational complexity is $O(I^{2})$. However, when employing spatial indexing structures such as KD-Tree or Ball Tree, the computational complexity for low-dimensional spatial point cloud datasets can be reduced to O(IlogI).

After the NELD-EC algorithm completes the initial neighborhood search for all data points, the EIP algorithm is employed to extract the valid neighborhood for each data point. The core component of the EIP algorithm is based on the DBSCAN algorithm. Considering that the computational complexity of DBSCAN is related to the number of elements in the clustered set and that the number of neighboring points varies across different data points, the average number of points within a neighborhood, denoted as ${\bar{I}}_{1}$, is used for computational complexity analysis. When performing a neighborhood search for the elements in the neighborhood distance set $D\left(p_{i}\right)$ of a given data point using DBSCAN, the computational complexity of brute-force searching is $O({\bar{I}}_{1}^{2})$, while that of using a spatial indexing structure is $O({\bar{I}}_{1}\log{\bar{I}}_{1})$. During cluster expansion in the DBSCAN algorithm, since minPts is set to 1, all points are treated as core points, leading to an additional computational complexity of $O({\bar{I}}_{1}^{2})$. The computational complexity of selecting valid clusters in the EIP algorithm is $O(\bar{I})$. Therefore, the overall computational complexity for eliminating interfering points within the neighborhood of a single data point is $O({\bar{I}}_{1}^{2})$. The total computational complexity involved in filtering the valid neighborhood set $S_{d}^{V}\left(p_{i}\right)$ for all data points in the point cloud dataset is $O(I\cdot {\bar{I}}_{1}^{2})$.

Defining the average number of data points in the valid neighborhood set as ${\bar{I}}_{2}$, the computational complexity of calculating the NELD for all data points in the NELD-EC algorithm is approximately $O(I\cdot {\bar{I}}_{2})$. When performing density clustering based on the NELD of all data points, the DBSCAN algorithm is utilized again, where the computational complexity of the neighborhood search is $O(I^{2})$ for brute-force searching or O(IlogI) when using a spatial indexing structure. The computational complexity of cluster expansion is $O(I^{2})$, resulting in an overall computational complexity of $O(I^{2})$ for the density clustering process.

The computational complexity introduced by defining the adaptive threshold $d_{i}^{+}$ for each data point in the NELD-EC algorithm is approximately O(I). The process of redefining the valid neighborhood set $S_{d}^{V}\left(p_{i}\right)$ based on the adaptive threshold $d_{i}^{+}$ involves a computational complexity of $O(I^{2})$ for brute-force searching or O(IlogI) when using a spatial indexing structure. Assuming that the average number of elements in the redefined valid neighborhood set $S_{d}^{V}\left(p_{i}\right)$ is ${\bar{I}}_{3}$, the computational complexity introduced by the cluster expansion step in the NELD-EC algorithm is $O(I\cdot {\bar{I}}_{3})$.

In summary, when the NELD-EC algorithm determines neighborhoods using a brute-force search approach, the computational complexity is $O(I^{2})$. Generally, the number of data points in a spatial point cloud, *I*, is significantly larger than the average number of points within a neighborhood. When a spatial indexing method is employed for neighborhood determination, the overall computational complexity is primarily influenced by the density clustering process. Consequently, under these conditions, the computational complexity of the NELD-EC algorithm remains $O(I^{2})$. However, when an excessively large initial distance threshold is used and the dataset consists of only a few clusters, the average number of neighborhood points, ${\bar{I}}_{1}$, ${\bar{I}}_{2}$, and ${\bar{I}}_{3}$, is no longer significantly smaller than the total number of data points *I* in the spatial point cloud. In this case, if the NELD-EC algorithm employs a spatial indexing approach for neighborhood determination, the computational complexity becomes $O(I\cdot \left(I+2\log\bar{I}+{\bar{I}}_{1}^{2}+{\bar{I}}_{2}+{\bar{I}}_{3}\right))$.

From the analysis of computational complexity, it can be observed that the density of a spatial point cloud or the density variation between clusters only affects the values of the average number of neighborhood points, ${\bar{I}}_{1}$, ${\bar{I}}_{2}$, and ${\bar{I}}_{3}$, but does not alter the complexity expression itself. From this perspective, it can also be inferred that the NELD-EC algorithm is capable of clustering not only point clouds with relatively uniform inter-cluster densities but also those exhibiting significant density variations among clusters.

The spatial complexity of the NELD-EC algorithm is primarily determined by the storage requirements for the neighborhood information of data points, resulting in a spatial complexity of O(I). Additionally, since the algorithm introduces a temporary storage set during the cluster expansion process, the extra spatial complexity incurred does not exceed O(I). Therefore, the overall spatial complexity of the NELD-EC algorithm remains O(I).

## 4. Experiments and Discussions

### 4.1. Experimental Preparation

In this section, the clustering performance of the NELD-EC algorithm after adopting the adaptive distance threshold on different datasets will be analyzed through several groups of experiments. The clustering algorithms involved in the comparative analysis include the classic EC algorithm [11], the EC algorithm with classification threshold (CFT-EC) [22], LDT-EC [13], and RDT-EC [14]. Among these algorithms, except for the CFT-EC algorithm, which requires dividing the point cloud intervals and allocating different distance thresholds to different intervals, and the RDT-EC algorithm, which needs to set an additional adjustment parameter, other algorithms only need to be given an initial distance threshold *d* before the experiments. To ensure the fairness of the comparison of the clustering performance of different algorithms, the initial distance threshold of each algorithm is selected as the value that makes the corresponding algorithm’s clustering performance optimal.

To quantitatively evaluate the clustering performance of different algorithms, referring to the F1 score, which is frequently employed as an index for evaluating classification performance in the field of machine learning [26], the macro F1 score is defined for evaluating the allocation effect of M clusters in point clouds. Similar to the definition of the F1 score, the macro F1 score is defined by the macro precision and macro recall rate. The macro precision is defined as

(16) $$P_{macro}=\frac{1}{M}\sum_{i=1}^{M}P_{i}$$

where $P_{i}$ represents the precision of the *i*-th cluster. The calculation of this parameter includes the number of data points that are misclassified as belonging to the i-th cluster, thus it can be used to analyze the false positive rate of that cluster. Similarly, the macro recall is defined as

(17) $$R_{macro}=\frac{1}{M}\sum_{i=1}^{M}R_{i}$$

with $R_{i}$ representing the recall of the *i*-th cluster. The calculation of recall includes not only the correctly clustered data points but also the incorrectly assigned data points. Therefore, recall can reflect the degree of misassigned data points in the cluster, which corresponds to the false negative rate. The macro precision $P_{macro}$ and macro recall $R_{macro}$ obtained from Equations (16) and (17) can be used to define the macro F1 score.

(18) $$F1_{macro}=\frac{2\times P_{macro}\times R_{macro}}{P_{macro}+R_{macro}}$$

the closer the macro F1 score is to 1, the more excellent the balance between the macro precision and the macro recall rate of the clustering algorithm, the stronger its ability to address under- and over-segmentation, and the better the comprehensive clustering performance of the clustering algorithm. Therefore, in this paper, the macro F1 score is used to comprehensively describe the situations of false positives and false negatives in point cloud clustering, thereby characterizing the clustering performance of the algorithm.

### 4.2. The Clustering Experiments Using Simulated Point Cloud Datasets

**Figure 4:**
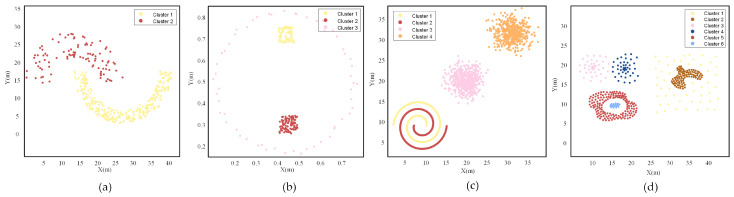
The point cloud distributions and truth labels of four public simulated datasets: (**a**) Jain. (**b**) Zelink6. (**c**) 2sp2glob. (**d**) Compound.

Firstly, the performance of the NELD-EC algorithm in processing irregular and non-uniform point clouds is analyzed by utilizing four public point cloud datasets: Jain, Zelink6, 2sp2glob, and Compound [27]. The distribution of point clouds in these datasets is shown in Figure 4, where point clouds of different colors represent different clusters. As shown in Figure 4, the Jain dataset consists of two interlaced crescent-shaped clusters with different densities; the Zelink6 dataset contains three clusters with different densities, two of which are located within a low-density annular cluster; the 2sp2glob dataset is composed of four clusters, two of which are intertwined helix-shaped clusters; and the Compound dataset consists of six clusters, including annular clusters, curved clusters, and densely packed clusters with non-uniform density, with some clusters exhibiting nested or adjacent distributions. Clearly, the datasets selected for the experiments all belong to irregular and non-uniform point cloud sets, which present significant challenges for point cloud clustering.

The four public point cloud datasets are processed using different clustering algorithms, with the parameters used by each algorithm shown in Table 1. The classical EC algorithm, LDT-EC algorithm, and NELD-EC algorithm require only the initial distance threshold to be set, whereas the RDT-EC and CFT-EC algorithms require two additional parameters. Both the RDT-EC and CFT-EC algorithms require calibration of the radar position. Additionally, the RDT-EC algorithm requires the setting of an adjustment parameter, while the CFT-EC algorithm requires the setting of the region arc radius. To compare the optimal performance of the different algorithms, the parameters of all algorithms were adjusted according to the specific characteristics of each dataset.

**Figure 5:**
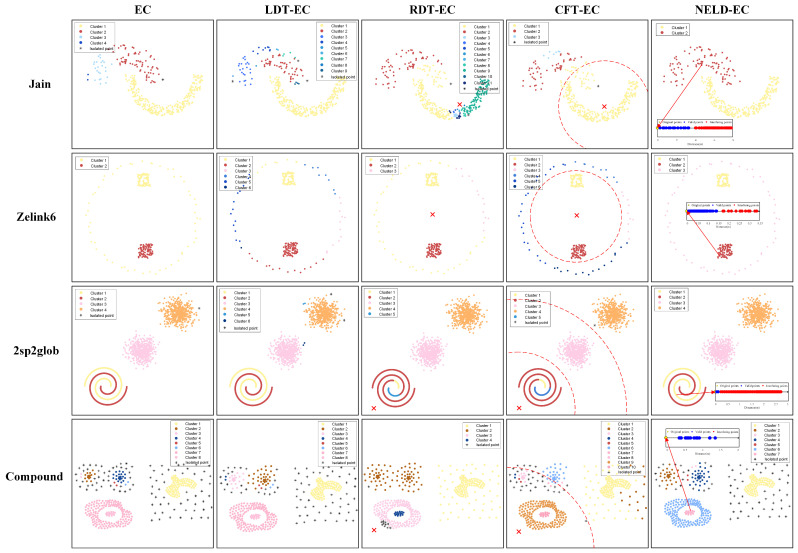
Clustering results of several algorithms on four public datasets.

Five clustering algorithms, using the parameters listed in Table 1, were applied to cluster the four-point cloud datasets shown in Figure 4. The resulting point cloud clustering outcomes are presented in Figure 5. In each subplot of Figure 5, different colors are used to represent different categories, with the legend indicating the number of clusters in each subplot. For data points that cannot be assigned to any category, a “*” symbol is used in the subplots of Figure 5. These points are identified as noise and can also be treated as a separate class.

The classical EC algorithm applied clustering to the four datasets with initial distance thresholds of 2.5 m, 0.07 m, 1.61 m, and 1 m, respectively. The corresponding clustering results are shown in the subplots of the first column of Figure 5. As can be seen from the results, although the EC algorithm selected the optimal initial distance threshold, over-segmentation occurred in the Jain, 2sp2glob, and Compound datasets, while under-segmentation occurred in the Zelink6 dataset. The fixed distance threshold approach used by the EC algorithm is unable to effectively cluster irregular and non-uniform point clouds.

The LDT-EC algorithm, which adjusts the distance threshold based on local density information, applied initial distance thresholds of 2.5 m, 0.08 m, 1.62 m, and 1.5 m for the four datasets, respectively. The corresponding clustering results are shown in the second column of each subplot in Figure 5. From the clustering results of the LDT-EC algorithm, it can be observed that severe over-segmentation occurred in the Jain and 2sp2glob datasets, with the number of clusters obtained being significantly higher than the actual number of clusters. When processing the Zelink6 dataset, the LDT-EC algorithm experienced over-segmentation within the annular cluster itself and under-segmentation within the two internal clusters, leading to numerous errors in the assignment of data points to clusters. When handling the more complex Compound dataset, the algorithm caused severe over-segmentation of the two circular clusters in the upper left part of the point cloud, with significant boundary effects, and under-segmentation occurred with the two nested point clouds in the lower left region, which were clustered as a single cluster. Therefore, similar to the classical EC algorithm, the LDT-EC algorithm is unable to handle such relatively complex point cloud data.

The RDT-EC algorithm adaptively adjusts the distance threshold by utilizing the relative position between data points and the radar, and the calculation formula for the distance threshold is presented in Equation (6). Since the RDT-EC algorithm requires computing the distance between data points and the radar during the clustering process, the positions of the radar in different datasets need to be specified. Considering the characteristic that the data collected by the radar shows a pattern of being denser near and sparser far, the two-dimensional positions of the radar selected in different datasets are (30, 8), (0.45, 0.5), (0, 0), and (10, 5), respectively, with the unit being meters. As a result, the minimum distances between data points and the lidar in the four datasets are 1.45 m, 0.16 m, 7.46 m, and 2.44 m. For different datasets, the initial distance thresholds chosen by the RDT-EC algorithm are 2.1 m, 0.08 m, 1 m, and 1.5 m, respectively, and the corresponding adjustment parameters are 0.15, 0.5, 0.8, and 0.15. Under the conditions of the above parameter settings, the clustering results of the RDT-EC algorithm for the four datasets are shown in the third column of each subplot in Figure 5, where the red “×” indicates the position of the lidar. From the clustering results of the RDT-EC algorithm, it can be observed that the number of clusters after clustering in the Jain dataset is significantly higher than the original two clusters. The root cause of this result lies in the fact that the Jain dataset does not possess the characteristic of being denser near and sparser far, thereby leading to a significant degradation in clustering performance. Under the reasonable setting of the radar position, the Zelink6 dataset exhibits the characteristic of being denser near and sparser far. Therefore, the RDT-EC algorithm performs relatively well in clustering the Zelink6 dataset, but there still exist issues of inaccurate data point allocation for the outer sparser clusters of this dataset. For the 2sp2glob dataset, the clustering effect of the two spiral clusters that are closer to the lidar is not satisfactory. The reason for this problem also lies in the insignificant characteristic of being denser near and sparser far for the data points in these two clusters. For the more complex Compound dataset, a relatively severe under-segmentation phenomenon occurs. Based on the above analysis, it can be seen that the point cloud clustering performance of the RDT-EC algorithm is closely related to the morphological characteristics of the point cloud, and its application has significant limitations.

The CFT-EC algorithm is also based on the characteristic of denser near and sparser far of data points. It divides data points into several regions according to their distances from the lidar and adopts different distance thresholds in different regions. Regarding the selection of the lidar position, the same setting as that of the RDT-EC algorithm can be adopted. The CFT-EC algorithm divides the Jain, Zelink6, and Compound datasets into two regions. The boundaries of the two regions are arcs centered at the lidar, with radii of 15.32 m, 0.25 m, and 15.80 m, respectively. The 2sp2glob dataset is divided into three regions, and the radii of the two boundary arcs are 16.67 m and 33.33 m, respectively. The distance thresholds within the two regions of the Jain dataset are 2 m and 3 m, respectively. The distance thresholds within the two regions of the Zelink6 dataset are 0.04 m and 0.06 m, respectively. The distance thresholds within the three regions of the 2sp2glob dataset are 0.5 m, 2 m, and 3 m, respectively. The distance thresholds within the two regions of the Compound dataset are 1m and 3m, respectively. Based on the above parameter settings, the CFT-EC algorithm is used to cluster the four datasets, and the corresponding results are shown in each subplot of the fourth column in Figure 5. The red “×” in the figure indicates the position of the lidar, and the red arc indicates the boundary between different regions. From the clustering results, it can be seen that the CFT-EC algorithm presents over-segmentation in the clustering process of the four datasets, indicating that this algorithm is also sensitive to the distribution characteristics of point clouds and has significant limitations in application.

The NELD-EC algorithm, which determines the adaptive distance threshold based on NELD, selects initial distance thresholds of 8 m, 0.34 m, 2.66 m, and 2 m for the four datasets, respectively. The corresponding clustering results are shown in the subgraphs of the last column of Figure 5. In each subgraph, a point located in the boundary region is selected, and the clustering results of its neighborhood distance set are displayed. After clustering the neighborhood distance set, blue points represent valid points, and red points represent interference points within the corresponding neighborhood distance set. From the clustering results of the neighborhood distance set selected from the data points in each subgraph, it can be seen that for points in the boundary region, the EIP algorithm can successfully distinguish valid points from interference points within the neighborhood, while grouping valid points together, thereby ensuring the performance of the subsequent clustering process. From the clustering results of the NELD-EC algorithm for the four datasets, it can be seen that the NELD-EC algorithm perfectly segmented the clusters of point clouds in the Jain, Zelink6, and 2sp2glob datasets, without over-segmentation or under-segmentation problems. For the more complex-shaped and density-varying Compound dataset, the NELD-EC algorithm is able to perfectly cluster the two nested point clouds in the lower-left region and the embedded point cloud on the right. However, the clustering of the two closely spaced circular point clouds in the upper-left region is not ideal, exhibiting some boundary effects. Additionally, for point clouds with lower density and data points on the edges of certain point clouds, clustering was not achieved, and they were identified as noise. Although the NELD-EC algorithm can handle point clouds with complex shapes and large density variations, it is still affected by boundary effects. Furthermore, this algorithm is unable to cluster extremely sparse point clouds. Compared to the other algorithms, the NELD-EC algorithm’s method of determining the distance threshold is more adaptable, requiring no special conditions on the structure of the point clouds, making it more capable of clustering irregular and non-uniform point clouds.

The clustering performance of each algorithm on the four datasets is further quantified using the corresponding macro F1 scores, based on the clustering results of the algorithms. The macro F1 scores of the various algorithms on the four datasets are shown in Table 2. As can be seen from Table 2, when the EC algorithm clusters the first three datasets, although it exhibits over-segmentation or under-segmentation issues, the number of misallocated data points is relatively low, resulting in a higher macro F1 score. Particularly, the EC algorithm’s clustering of the 2sp2glob dataset has a very small proportion of misclassified points, with the macro F1 score being 0.99, which is very close to a perfect clustering result. The LDT-EC algorithm performs poorly on the Jain and Zelink6 datasets, with corresponding macro F1 scores around 0.85, while its clustering performance on the 2sp2glob dataset is comparable to that of the EC algorithm, yielding a macro F1 score of 0.99. Since none of the four datasets in the experiment exhibit a clear near-dense-far-sparse pattern, both the RDT-EC and CFT-EC algorithms yield lower macro F1 scores on all four datasets, with the lowest score for the RDT-EC algorithm applied to the Jain dataset, around 0.69. For the NELD-EC algorithm, the macro F1 scores for the first three datasets are all 1, indicating perfect clustering on these three datasets. For the more complex point cloud structure of the Compound dataset, all algorithms yield low macro F1 scores, but the NELD-EC algorithm shows significantly better performance than the other algorithms.

By comparing the clustering results of several algorithms on the Jain, Zelink6, 2sp2glob, and Compound datasets, it can be observed that the RDT-EC and CFT-EC algorithms require a larger number of adjustable parameters and are highly dependent on the distribution characteristics of the point clouds, resulting in limited applicability. In contrast, the EC, LDT-EC, and NELD-EC algorithms only require the initial distance threshold to be set, making parameter adjustment relatively simple. However, when dealing with more complex point cloud data, both the EC and LDT-EC algorithms suffer from significant over-segmentation or under-segmentation issues. The NELD-EC algorithm, which utilizes NELD, better reflects the local density of point clouds. The adaptive distance threshold determined based on this can more effectively reduce the occurrence of over-segmentation or under-segmentation, enabling efficient clustering of irregular and non-uniform point clouds.

### 4.3. The Experiments Using the Fixed-Point Collected Dataset

**Figure 6:**
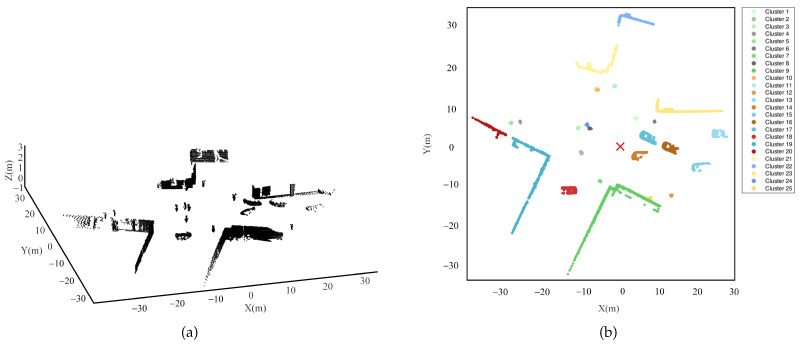
Point cloud distribution of the fixed-point collected dataset: (**a**) 3D distribution of the unlabeled point cloud. (**b**) Projection of the labeled point cloud on the horizontal plane.

To further validate the clustering performance of the NELD-EC algorithm proposed in this paper, a comparative analysis was carried out using the Sydney Urban Objects dataset [28]. The Sydney Urban Objects dataset is a typical fixed-point collected dataset, which consists of point clouds of common urban road scenes collected by the mechanical lidar Velodyne HDL-64E LIDAR in the Central Business District of Sydney, Australia. A certain frame of data from this dataset was selected as the experimental data. After eliminating the data points reflecting the ground, the corresponding three-dimensional point cloud is shown in Figure 6a,b, which shows the projection of this point cloud on the horizontal plane. The origin of the point cloud data is the position of the lidar, indicated by the red “×”. Through the analysis of the experimental data, it was determined that the data contained 25 clusters. The forms and annotations of each cluster are shown in Figure 6b. As can be seen from Figure 6b, the annotated targets involve vehicles, pedestrians, buildings, etc.

The experimental data originated from the scanning of real scenes by a lidar. Due to the varying distances between the target and the lidar, the point cloud data exhibit distinct density variances, and the relative positional relationships among different clusters are also random. Hence, this experimental data can be utilized to assess the performance of clustering algorithms when processing real-scene data, particularly the clustering capability in dealing with point clouds with remarkable density differences [27]. This experiment is designed to evaluate the effectiveness of the NELD-EC algorithm in segmenting actual point cloud data and compare the sensitivity of this algorithm to parameter selection with other similar algorithms.

The EC, LDT-EC, RDT-EC, CFT-EC, and NELD-EC algorithms were applied to cluster the experimental data shown in Figure 6. The parameter settings for each algorithm are presented in Table 3. The parameters for each algorithm in Table 3 were tuned based on the experimental data.

**Figure 7:**
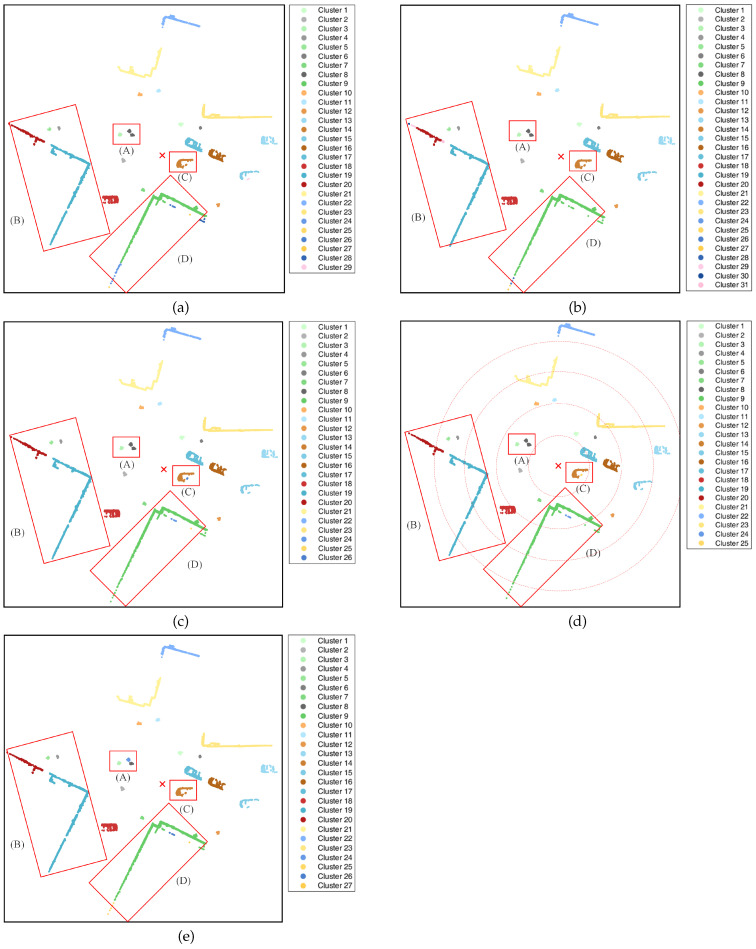
The clustering results of the fixed-point collected dataset by several algorithms: (**a**) EC. (**b**) LDT-EC. (**c**) RDT-EC. (**d**) CFT-EC. (**e**) NELD-EC.

Based on the parameters in Table 3, the clustering results for the point cloud data shown in Figure 6 were obtained using each clustering algorithm, as illustrated in the subgraphs of Figure 7. In each subgraph of Figure 7, the red “×” marks the position of the lidar. To visually compare the clustering performance of different algorithms, four identical regions, denoted as Region (A), Region (B), Region (C), and Region (D), are marked with red boxes in the clustering results of the different algorithms, as shown in the subgraphs of Figure 7. The four selected regions for comparison are all areas with higher clustering difficulty.

The classical EC algorithm selects an initial distance threshold of 1m, and the corresponding clustering results are presented in Figure 7a. In contrast to Figure 6b, the EC algorithm achieves relatively better clustering for regions (B) and (C). Nevertheless, the EC algorithm fails to completely separate the three pedestrians in region (A) and erroneously clusters two of them into one cluster, giving rise to an under-segmentation phenomenon. Regarding region (D), the buildings have a considerable span, and the corresponding point cloud exhibits a distribution characteristic of being “denser near and sparser far”. For the EC algorithm with a fixed distance threshold, the point cloud at the far end of the building is segmented into multiple clusters, leading to a severe over-segmentation phenomenon. Although the EC algorithm selects the optimal initial distance threshold, due to its fixed nature, it is challenging to adapt to the point cloud data with non-uniform density and random position distribution in real road scenes and thus fails to conduct effective clustering processing for complex scenarios.

In this dataset, the LDT-EC algorithm performs clustering on the experimental data according to the initial distance threshold of 1.4 m, and the corresponding clustering results are presented in Figure 7b. In comparison with Figure 7a, the LDT-EC algorithm has similar issues as the EC algorithm in regions (A) and (D). At the same time, the LDT-EC algorithm presents a severe over-segmentation problem in region (B). Evidently, although the LDT-EC algorithm adopts an adaptive distance threshold, its clustering effect is suboptimal when handling datasets with significant density variances.

The RDT-EC algorithm adaptively adjusts the distance threshold by exploiting the relative position between the data points and the lidar. In the experiment, the initial distance threshold of this algorithm was selected as 1m, and the adjustment parameter was 0.3. Based on the positional information of the lidar and the distribution information of the dataset, it is known that the minimum distance between the data points and the lidar is 4.23 m. Under the above parameter configuration, the clustering results of the RDT-EC algorithm are depicted in Figure 7c. In contrast to Figure 7a,b, the clustering in region (A) presents the same issues as those in the EC algorithm and the LDT-EC algorithm. Regarding the point cloud clustering in regions (B) and (D), since the point clouds in these two regions exhibit the feature of being dense in proximity and sparse in the distance, which conforms to the application scenario of the RDT-EC algorithm, far superior clustering effects are achieved compared to the EC algorithm and the LDT-EC algorithm. Nevertheless, for region (C), as this region is relatively close to the radar, the corresponding adaptive distance threshold is relatively small. When clustering the point cloud of a single vehicle, due to the dispersion of some point cloud data, it is divided into two clusters, resulting in an over-segmentation phenomenon.

The CFT-EC algorithm divides the point cloud data into five regions based on their distances from the radar, where the radii of the four demarcating arcs are 7.41 m, 14.82 m, 22.23 m, and 29.64 m, respectively. The distance thresholds for the five regions, from the nearest to the farthest, are set as 1 m, 2 m, 3 m, 4 m, and 5 m respectively. After the experimental data are clustered by the CFT-EC algorithm, the corresponding results are depicted in Figure 7d. The four concentric circular arcs in Figure 7d are the boundaries of the different regions. When comparing Figure 7d with Figure 7c, the clustering effects of the point clouds in the four concerned regions are comparable to those of the RDT-EC algorithm. Both algorithms can achieve relatively good clustering for regions (B) and (D). However, for regions (A) and (C) that are closer to the lidar, they fail to cluster correctly, presenting either over- or under-segmentation.

The NELD-EC algorithm proposed in this paper sets the initial distance threshold at 1.4 m, and the clustering results of the experimental data are depicted in Figure 7e. As can be seen from Figure 7e, the NELD-EC algorithm effectively segments the point clouds in regions (A), (B), and (C), but there exists a certain degree of over-segmentation in the point cloud of region (D). Observing the point cloud distribution and segmentation results in region (D), it can be noticed that the NELD-EC algorithm exhibits over-segmentation when clustering the point clouds in regions with a relatively sparse distribution. The cause of this issue lies in the relatively small adaptive distance threshold in this region. In comparison with the clustering results of several other algorithms, the NELD-EC algorithm can achieve perfect segmentation of region (A), which cannot be effectively clustered by other algorithms. For regions (B) and (C), the NELD-EC algorithm can also attain the optimal clustering effect. Only when it comes to region (D), the clustering performance of the NELD-EC algorithm slightly deteriorates, but it is still superior to the EC algorithm and the LDT-EC algorithm.

Through the analysis of the clustering results presented in each subfigure of Figure 7, it can be observed that the clustering effects of different algorithms on the point clouds in regions (A), (B), (C), and (D) vary, as presented in Table 4. As is evident from Table 4, for the point cloud in region (D), none of the algorithms can achieve effective segmentation. However, for the other three regions, only the NELD-EC algorithm is capable of achieving accurate segmentation, while the other algorithms can only achieve effective segmentation for specific regions.

Further, the clustering performance of different algorithms was analyzed quantitatively. The macro F1 scores of the corresponding algorithms were calculated based on the clustering results of each subplot in Figure 7, as presented in Table 5. It can be observed from Table 5 that the macro F1 scores of several algorithms all exceed 0.9, suggesting that these algorithms can assign data points in the scene to their respective clusters with relatively high accuracy. The macro F1 score of the NELD-EC algorithm is the highest, reaching 0.955, indicating that when processing the point cloud data collected at a fixed point, this algorithm has the least misassigned data points, and the clusters partitioned are more in line with the actual situation.

To further analyze the impact of uncertainty in the experimental point cloud on the clustering results, the resampling-based Bootstrap method was used to randomly sample 80% of the data from the fixed-point sampling dataset, with 10 sets of data randomly selected. Different clustering algorithms were then applied to these 10 sets of data, and the 95% confidence intervals of the macro F1 scores for each algorithm were compared based on the clustering results, as shown in Table 6. Table 6 also provides the average macro F1 score for each clustering algorithm, based on the 10 sets of clustering results. As shown in Table 6, the NELD-EC algorithm has the highest average macro F1 score of 0.943, while the CFT-EC algorithm has the lowest average macro F1 score of 0.878. Comparing the 95% confidence intervals for each algorithm, the NELD-EC algorithm is in the higher range, with a narrower interval. The confidence interval analysis based on the resampling Bootstrap method shows that the NELD-EC algorithm has a higher and more stable clustering performance.

As previously stated, the clustering performance of common EC-type algorithms is highly sensitive to the initial distance threshold. However, the NELD-EC algorithm proposed herein is insensitive to the initial distance threshold, thereby making the NELD-EC algorithm more applicable in engineering. To validate this conclusion, the point cloud dataset depicted in Figure 6 is still taken as the research object to investigate the impact of the initial distance threshold on the macro F1 score. Due to the fact that the CFT-EC algorithm has initial distance thresholds for multiple regions, which is different from the single initial distance threshold employed by other algorithms, the CFT-EC algorithm is not suitable for analyzing the influence of the initial distance threshold on the macro F1 score. Therefore, in this experiment, the influence of the initial distance threshold on the macro F1 score of the CFT-EC algorithm is not compared.

**Figure 8:**
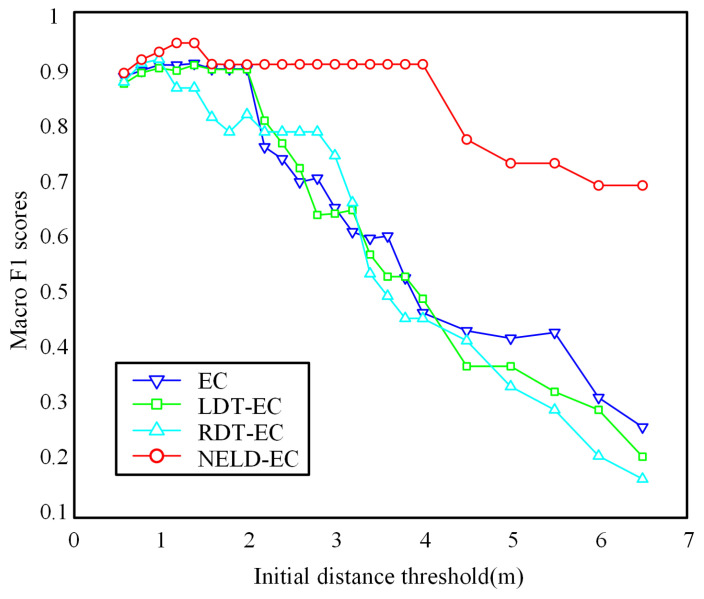
The impact of the initial distance threshold on the clustering performance of different algorithms.

The variations in the macro F1 scores of the EC algorithm, the LDT-EC algorithm, the RDT-EC algorithm, and the NELD-EC algorithm for the clustering results of the experimental data when the initial distance threshold varies within the range of 0.6 m to 6.5 m are depicted in Figure 8.

It can be observed from Figure 8 that the variation patterns of the macro F1 scores of the EC algorithm and the LDT-EC algorithm are largely consistent. When the initial distance threshold is selected to be lower than 2 m, both algorithms maintain relatively high macro F1 scores, presenting a superior clustering effect. When the initial distance threshold is higher than 2 m, with the increase in the initial distance threshold, the macro F1 scores of both algorithms keep decreasing continuously. The macro F1 score of the RDT-EC algorithm is more significantly affected by the initial distance threshold, maintaining a high macro F1 score only within a relatively small region, and then it keeps decreasing along with the increase in the initial distance threshold. At higher initial distance thresholds, the macro F1 scores of the EC algorithm, the LDT-EC algorithm, and the RDT-EC algorithm are basically equivalent. The macro F1 score of the NELD-EC algorithm remains at a high level within a wide range of initial distance thresholds. Only when the initial distance threshold is higher than 4 m, does the macro F1 score decline slowly, but it is still much higher than the macro F1 scores of the other three algorithms. Thus, it can be concluded that the clustering performance of the NELD-EC algorithm is insensitive to the selection of the initial distance threshold.

Further analysis was conducted on the effect of initial distance threshold variations in the 95% confidence interval of the macro F1 score. Based on the macro F1 scores of each algorithm shown in Figure 8, the corresponding 95% confidence intervals of the macro F1 scores were calculated, as shown in Table 7. For the urban intersection scene shown in Figure 6, when the initial distance threshold varies between 0.5 m and 6.5 m, the 95% confidence interval for the macro F1 score of the NELD-EC algorithm is [0.8407, 0.9088]. Compared with other clustering algorithms, the 95% confidence interval of the macro F1 score for the NELD-EC algorithm is clearly in a higher range with a narrower interval. This indicates that the clustering performance of the NELD-EC algorithm is relatively insensitive to the selection of the initial distance threshold, and within a wide range of initial distance thresholds, the NELD-EC algorithm is able to maintain a high and stable macro F1 score.

**Figure 9:**
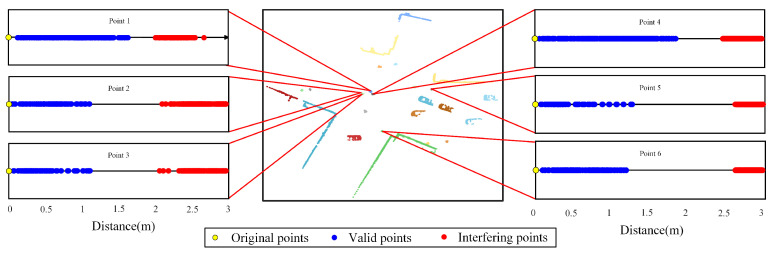
Detection of the interfering points in the neighborhood distance set.

Based on the clustering results depicted in Figure 7e, a further analysis is conducted on the reason why the clustering performance of the NELD-EC algorithm is not prominently influenced by the initial distance threshold. In the experimental outcomes, six data points are selected and labeled as Point 1, Point 2, Point 3, Point 4, Point 5, and Point 6. When the initial distance threshold is 3 m, the distribution of elements in the neighborhood distance set of each data point on the distance axis is obtained, as shown in Figure 9. On the distance axis of each data point shown in Figure 9, the yellow point serves as the origin of the distance axis and is also the position where the data point is located. The blue and red points, respectively, indicate the valid neighborhood points and interference points detected by Algorithm 1.

It can be observed from Figure 9 that, under the given initial distance threshold condition, there exist interfering points within the neighborhood of the data points. However, these interfering points present a distinct clustering phenomenon with the valid points in terms of distance, where the two types of data points can be distinguished and the interfering points can be eliminated through simple clustering processing. For the initial distance threshold with a wide range of fluctuations, since the interfering points introduced by the change in the neighborhood range can all be identified and removed, the influence of the initial distance threshold on the numerical value of the local density of the data points is weakened. This further enables the clustering performance of the NELD-EC algorithm to remain stable within a large range of values of the initial distance threshold.

Through the analysis of the clustering results of the fixed-point collection dataset, it can be observed that the NELD-EC algorithm can effectively identify and eliminate the interfering points within the neighborhood of the data point. Hence, this algorithm is insensitive to the initial distance threshold. Consequently, the NELD-EC algorithm can maintain a relatively high clustering performance within a broad range of initial distance threshold values. Simultaneously, the NELD-EC algorithm can effectively eliminate the interfering points within the neighborhood of the data points, and the local density calculated by this algorithm can better reflect the true local information of the data point. The adaptive distance threshold determined by the local density of all the data points within the data set is more rational, thereby the NELD-EC algorithm possesses a more superior clustering performance.

### 4.4. The Experiments Using the Sequential Point Cloud Dataset

To validate the capability of the NELD-EC algorithm in handling sequence point clouds, the publicly available KITTI Odometry dataset [2] was adopted. The KITTI Odometry dataset is currently the largest dataset for evaluating a computer vision algorithm in the context of autonomous driving scenarios worldwide. It encompasses video data captured by cameras in both color and grayscale, as well as point cloud data collected by lidar. In this part, only the sequential point cloud data captured by lidar within this dataset is utilized for analysis. The total driving distance of the data acquisition platform amounts to 39.2 km, with 10 frames collected per second, resulting in a cumulative total of 41,000 frames. These frames are classified into 22 sequences based on the varying collection scenarios. The point cloud data of each frame within the KITTI Odometry dataset is presented in the vehicle coordinate system, where the lidar serves as the origin, the forward direction parallel to the vehicle body in the horizontal plane is the positive X-axis, the direction perpendicular to the vehicle body and pointing to the left in the horizontal plane is the positive Y-axis, and the upward direction is the positive Z-axis. The point cloud data within the KITTI Odometry dataset encompasses various targets such as automobiles, buildings, and trees. Jens Behley et al. have annotated various targets within the dataset [29]. In this paper, 3000 frames from sequence #00 and 1000 frames from sequence #10 of the KITTI Odometry dataset were, respectively, selected as the sequential point cloud dataset for this part. Frame-by-frame clustering processing on the sequential point cloud dataset was conducted, and the macro F1 score was calculated for each frame.

**Figure 10:**
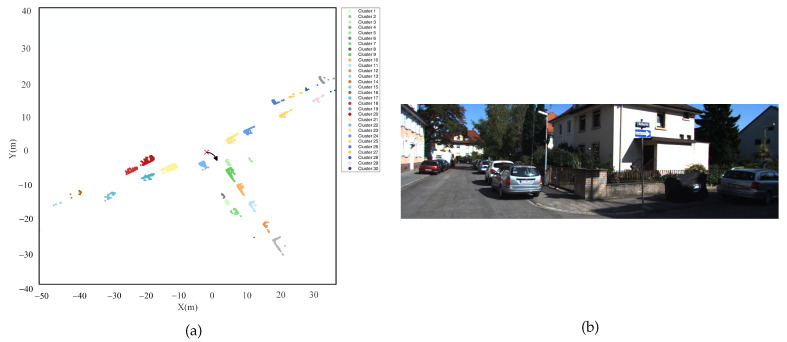
The 2694th frame of Sequence #00: (**a**) Projection of the annotated point cloud on the horizontal plane. (**b**) Optical image in front of the acquisition platform.

The data of Sequence #00 were collected on urban road sections, where the environment is rather complex and the vehicle density is relatively high. Figure 10 presents the annotated point cloud and the corresponding optical image of the 2694th frame of Sequence #00. Figure 10a depicts the horizontal projection of the point cloud data of this frame, where the red “×” indicates the position of the LiDAR, and the black arc arrow represents the turning direction of the acquisition platform. Figure 10b shows the optical image captured by the forward camera of the acquisition platform. As can be seen from Figure 10b, a large number of vehicles are parked on both sides of the road. Within the effective detection range of the LiDAR, as the distance varies, there are considerable differences in the point cloud density and distribution patterns of different vehicles, as shown in Figure 10a.

**Figure 11:**
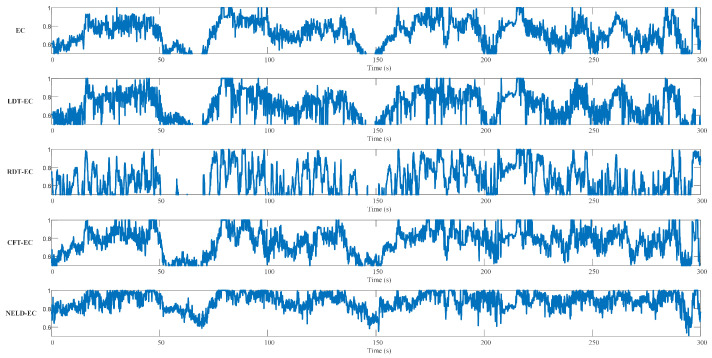
The temporal variations in the macro F1 scores of the clustering results for each algorithm in each frame of sequence #00.

To investigate the clustering efficacy of different algorithms when handling sequential point cloud data, the initial distance thresholds of the EC algorithm, LDT-EC algorithm, RDT-EC algorithm, and NELD-EC algorithm were all set at 3 m, and the adjustment parameter of the RDT-EC algorithm was set at 0.3. Meanwhile, based on the effective range of the LiDAR, the monitoring area of the CFT-EC algorithm was uniformly divided into five regions, with the distance thresholds of each region set at 2 m, 2.5 m, 3 m, 3.5 m, and 4 m, respectively. For sequence #00, each frame of data can be treated as an experimental sample. Based on the above parameter settings, each frame of sequence #00 was clustered by each algorithm, and the macro F1 score corresponding to the clustering result of that frame was computed. The temporal variations in the macro F1 scores of different algorithms are shown in Figure 11.

It can be observed from Figure 11 that, with the change in time, the macro F1 scores of the five algorithms present obvious fluctuations over time. Particularly around 70 s, 150 s, and 290 s, the macro F1 scores of all the algorithms decrease significantly, suggesting that the point clouds at the corresponding positions are not easy to segment, there are numerous misallocations of data points, and the clustering performance of the algorithms deteriorates. The macro F1 scores of the EC algorithm, LDT-EC algorithm, and RDT-EC algorithm also show a marked reduction around 200 s, while the macro F1 scores of the CFT-EC algorithm and NELD-EC algorithm do not change significantly at this region, indicating that the CFT-EC algorithm and NELD-EC algorithm have superior point cloud clustering performance at this location. Considering the entire time variation interval, the fluctuation of the macro F1 score of the RDT-EC algorithm is more intense, and the fluctuation range of the macro F1 score of the NELD-EC algorithm is the smallest. The results in Figure 11 demonstrate that, under the condition of a fixed initial threshold, the macro F1 score of the NELD-EC algorithm is more stably maintained within a higher performance range.

To quantitatively analyze the performance of different clustering algorithms, statistical analysis was conducted on the macro F1 score arrays obtained from sequence #00 for each algorithm, as shown in Table 8. Table 8 compares the mean, standard deviation, correlation time, maximum value, minimum value, and 95% confidence interval of the macro F1 scores for each algorithm. The optimal values for each parameter are highlighted in blue in Table 8. As seen in Table 8, the NELD-EC algorithm has the highest mean macro F1 score, 0.878, while the classical EC algorithm and CFT-EC algorithm have slightly lower mean values, and the LDT-EC algorithm and RDT-EC algorithm have the lowest macro F1 scores. In terms of standard deviation, correlation time, and confidence interval, the NELD-EC algorithm has the smallest standard deviation, the longest correlation time, and the narrowest confidence interval, indicating that the macro F1 score of the NELD-EC algorithm has the least fluctuation, and thus, the clustering performance of the NELD-EC algorithm is the most stable. All algorithms reach a maximum macro F1 score of 1, but there are significant differences in the minimum macro F1 scores, with the minimum macro F1 score of the NELD-EC algorithm being 0.507, much higher than the corresponding values of the other algorithms. Through statistical analysis, it can be seen that when processing sequence #00, the NELD-EC algorithm demonstrates higher and more stable clustering performance.

**Figure 12:**
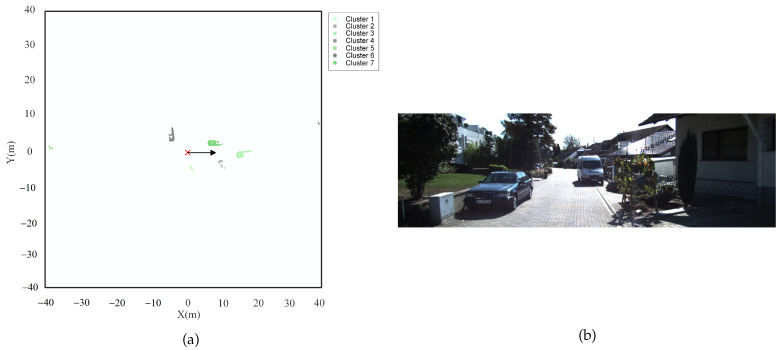
The 352nd frame of Sequence #10: (**a**) Projection of the annotated point cloud on the horizontal plane. (**b**) Optical image in front of the acquisition platform.

Herein, the ability of different algorithms in handling sequence point cloud is further analyzed using sequence #10 from the KITTI Odometry dataset. Sequence #10 was collected in a suburban section close to a highway, mainly featuring straight paths and a low vehicle density. Figure 12 presents the annotated point cloud and the corresponding optical image of frame 352 in sequence #10. Figure 12a shows the projection of the point cloud of this frame on the horizontal plane, where the red “×” represents the position of the lidar, and the black arrow indicates the forward direction of the acquisition platform. Figure 12b is the optical image captured by the forward camera of the acquisition platform. In comparison with Figure 10a, there are fewer vehicle targets in Figure 12a, and the point clouds of these vehicles exhibit different degrees of sparsity as the distance from the lidar varies.

**Figure 13:**
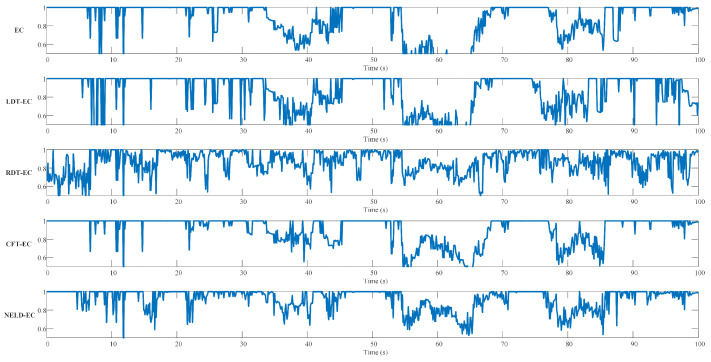
The temporal variations in the macro F1 scores of the clustering results for each algorithm in each frame of sequence #10.

The parameters of each algorithm are adopted with the same values as those when processing sequence #00. The sequence #10 is subjected to frame-by-frame clustering processing, and the macro F1 score of the clustering result of each frame is calculated. The temporal variations in the macro F1 scores of different algorithms are depicted in Figure 13.

As can be observed from Figure 13, the macro F1 scores of several algorithms exhibited significant fluctuations within the range of 30 s to 90 s, suggesting that the road conditions during this period were rather complex, and the clustering performance of several algorithms declined. During the period from 55 s to 65 s, the clustering performance of all algorithms decreased conspicuously. Among them, the performance of the EC algorithm and the LDT-EC algorithm dropped most significantly, the performance degradation of the RDT-EC algorithm was the least, and the performance of the NELD-EC algorithm was slightly lower than that of the RDT-EC algorithm but superior to that of the other algorithms. Throughout the entire experimental interval, the macro F1 scores of the EC algorithm, the CFT-EC algorithm, and the NELD-EC algorithm remained at a high level for a longer duration. However, when dealing with point cloud clustering in complex road conditions, although the macro F1 score of the NELD-EC algorithm also decreased, the decrease was not as significant as that of the other two algorithms.

After clustering sequence #10, the statistical parameters of the macro F1 scores for several algorithms are shown in Table 9. As seen in Table 9, all the statistical parameters of the macro F1 score for the NELD-EC algorithm reach the optimal values. Therefore, compared to the other typical algorithms, the NELD-EC algorithm is more suitable for clustering sequence point cloud data in practical scenarios.

Through the comparison of the processing results of two different sequence point clouds in the KITTI Odometry dataset by different algorithms, under the fixed initial distance threshold condition, the NELD-EC algorithm is capable of maintaining a relatively high clustering performance. As the surrounding environment of the acquisition platform varies, the clustering performance of the NELD-EC algorithm undergoes some fluctuations, but the fluctuation range is small and the stability is excellent. Therefore, the NELD-EC algorithm is more suitable for processing sequential point cloud data collected from real-world scenarios.

## 5. Conclusions and Outlook

Regarding the clustering issue of point clouds with irregular shapes and non-uniform densities, a novel Euclidean clustering algorithm is proposed in this paper and is named the NELD-EC algorithm. The NELD-EC algorithm calculates the local density of data points based on the NELD (Neighborhood Effective Local Density) of effective points within the neighborhood of the data point. By using the NELD of data points, it can more accurately describe the point cloud distribution characteristics around the data point. Based on the NELD of all data points, the NELD-EC algorithm provides a method to determine the adaptive distance threshold. Utilizing this adaptive distance threshold can effectively reduce the possibility of over-segmentation and under-segmentation, allowing the NELD-EC algorithm to effectively cluster point clouds with irregular shapes and non-uniform densities.

The results of comparative experiments on clustering using simulated point clouds, point clouds collected at fixed points, and sequential point clouds indicate that, in comparison with other classical EC-type algorithms, the clustering performance of the NELD-EC algorithm is insensitive to the selection of the initial distance threshold and has no specific requirements regarding the structure of the point clouds to be processed. When dealing with sequential point clouds, the NELD-EC algorithm can maintain a relatively high clustering performance, with a small fluctuation range and strong stability. The experimental results demonstrate that the parameter setting of the NELD-EC algorithm is straightforward and it can adaptively handle point clouds with complex structures, making it applicable for clustering data collected in actual scenarios.

The NELD-EC algorithm is a distance-based clustering algorithm, which is inevitably affected by boundary effects. The strength of the boundary effect is also related to the density differences between clusters. A more detailed statistical analysis of the NELD-EC algorithm requires exploring methods to reduce the boundary effect, which will be one of the key areas for future research.

In this paper, when processing sequential point clouds, the NELD-EC algorithm performs clustering on each frame individually. This method neglects the correlation between frames in the sequential point clouds, resulting in redundant calculations during clustering, which reduces the real-time performance of the algorithm when processing sequential point clouds. Utilizing the correlation between consecutive frames of the sequential point clouds, or incorporating semantic information [30,31], will help improve the precision and efficiency of the NELD-EC algorithm in processing sequential point clouds. Future research can focus on these aspects.

The NELD-EC algorithm effectively clusters point clouds with relatively complex structures, but there is still considerable room for improvement in terms of both performance and applicability. It may be considered to combine it with other algorithms, such as differential entropy [32], to improve the detection of interference points within the neighborhood of data points, enhancing the clustering performance of the NELD-EC algorithm and extending its applicability to a wider range of point clouds.

## Figures and Tables

**Table 1:** Parameter settings for clustering algorithms on four public datasets.

Algorithm	Parameters	Jain	Zelink6	2sp2glob	Compound
EC	Initial Distance Threshold (m)	2.5	0.07	1.61	1
LDT-EC	Initial Distance Threshold (m)	2.5	0.08	1.62	1.5
RDT-EC	Radar Position (m)	(30, 8)	(0.45, 0.5)	(0, 0)	(10, 5)
	Initial Distance Threshold (m)	2.1	0.08	1	1.5
	Adjustment Parameter	0.15	0.5	0.8	0.15
CFT-EC	Radar Position (m)	(30, 8)	(0.45, 0.5)	(0, 0)	(10, 5)
	Arc Radius of Region (m)	15.32	0.25	16.67, 33.33	15.80
	Initial Distance Threshold (m)	2, 3	0.04, 0.06	0.5, 2, 3	1, 3
NELD-EC	Initial Distance Threshold (m)	8	0.34	2.66	2.0

**Table 2:** Macro F1 scores of clustering results for several algorithms on four public datasets (optimal values are highlighted in blue).

Algorithm	Jain	Zelink6	2sp2glob	Compound
EC	0.91	0.89	0.99	0.75
LDT-EC	0.86	0.83	0.99	0.78
RDT-EC	0.69	0.81	0.82	0.65
CFT-EC	0.84	0.88	0.83	0.77
NELD-EC	1.00	1.00	1.00	0.81

**Table 3:** Parameter settings for clustering the fixed-point collection dataset using different algorithms.

Algorithm	Parameters	Value
EC	Initial Distance Threshold (m)	1
LDT-EC	Initial Distance Threshold (m)	1.4
RDT-EC	Initial Distance Threshold (m)	1
	Adjustment Parameter	0.3
CFT-EC	Arc Radius of Region (m)	7.41, 14.82, 22.23, 29.64
	Initial Distance Threshold (m)	1, 2, 3, 4, 5
NELD-EC	Initial Distance Threshold (m)	1.4

**Table 4:** The clustering effects of several algorithms on point clouds of different regions.

Algorithm	Region (A)	Region (B)	Region (C)	Region (D)
EC	Under-Segmentation	Accurate Segmentation	Accurate Segmentation	Over- and Under-Segmentation
LDT-EC	Under-Segmentation	Over-Segmentation	Accurate Segmentation	Over- and Under-Segmentation
RDT-EC	Under-Segmentation	Accurate Segmentation	Over-Segmentation	Over- and Under-Segmentation
CFT-EC	Under-Segmentation	Accurate Segmentation	Over-Segmentation	Over- and Under-Segmentation
NELD-EC	Accurate Segmentation	Accurate Segmentation	Accurate Segmentation	Over- and Under-Segmentation

**Table 5:** The macro F1 scores of the clustering results of the fixed-point collected dataset by several algorithms (optimal values are highlighted in blue).

Algorithm	Macro F1 Scores
EC	0.910
LDT-EC	0.909
RDT-EC	0.911
CFT-EC	0.911
NELD-EC	0.955

**Table 6:** Statistical parameters of stability for clustering results of each algorithm on multiple sampled datasets.

Algorithm	Mean	Confidence Interval
EC	0.907	[0.8939, 0.9209]
LDT-EC	0.909	[0.9073, 0.9103]
RDT-EC	0.902	[0.8921, 0.9110]
CFT-EC	0.878	[0.8608, 0.8945]
NELD-EC	0.943	[0.9329, 0.9521]

**Table 7:** Results of the perturbation of the initial distance threshold on the macro F1 score confidence interval of the clustering algorithms.

Algorithm	Confidence Interval
EC	[0.5812, 0.7576]
LDT-EC	[0.5580, 0.7504]
RDT-EC	[0.5409, 0.7404]
NELD-EC	[0.8407, 0.9088]

**Table 8:** The statistical parameters of macro F1 scores corresponding to different algorithms on sequence #00 (optimal values are highlighted in blue).

Algorithm	Mean	Standard Deviation	Correlation Time	Maximum	Minimum	Confidence Interval
EC	0.717	0.14137	144.38	1	0.302	[0.7123, 0.7217]
LDT-EC	0.692	0.16624	141.77	1	0.203	[0.6861, 0.6979]
RDT-EC	0.623	0.19692	136.34	1	0.212	[0.6160, 0.6300]
CFT-EC	0.754	0.13999	144.98	1	0.344	[0.7490, 0.7590]
NELD-EC	0.878	0.09086	148.36	1	0.507	[0.8747, 0.8813]

**Table 9:** The statistical parametersof macro F1 scores corresponding to different algorithms on sequence #10 (optimal values are highlighted in blue).

Algorithm	Mean	Standard Deviation	Correlation Time	Maximum	Minimum	Confidence Interval
EC	0.877	0.18646	47.78	1	0.303	[0.8513, 0.8647]
LDT-EC	0.861	0.19213	47.57	1	0.326	[0.8541, 0.8679]
RDT-EC	0.858	0.12187	48.97	1	0.333	[0.8536, 0.8624]
CFT-EC	0.904	0.14628	48.67	1	0.326	[0.8988, 0.9092]
NELD-EC	0.918	0.10396	49.19	1	0.486	[0.9143, 0.9217]

## Data Availability

Data are contained within the article.

## Abbreviations

The following abbreviations are used in this manuscript:

EC	Euclidean Clustering
LDT	Local Density-based Threshold
LDT-EC	LDT-based Euclidean Clustering
RDT	Relative Distance-based Threshold
RDT-EC	RDT-based Euclidean Clustering
NELD	Neighborhood Effective Line Density
NELD-EC	NELD-based Euclidean Clustering
KNN	K-Nearest Neighbors
RNN	Reverse K-Nearest Neighbors
DBSCAN	Density-based Spatial Clustering of Applications with Noise
CFT	Classification Threshold
CFT-EC	CFT-based Euclidean Clustering
KITTI	Karlsruhe Institute of Technology and Toyota Technological Institute at Chicago
